# A Testing Method for Shipborne Atomic Gravimeter Based on the Modulated Coriolis Effect

**DOI:** 10.3390/s23020881

**Published:** 2023-01-12

**Authors:** Yin Zhou, Can Zhang, Peijun Chen, Bing Cheng, Dong Zhu, Kainan Wang, Xiaolong Wang, Bin Wu, Zhongkun Qiao, Qiang Lin, Rui Li

**Affiliations:** 1 Zhejiang Provincial Key Laboratory of Quantum Precision Measurement, College of Science, Zhejiang University of Technology, Hangzhou 310023, China; 2 Institute for Frontiers and interdisciplinary Sciences, Zhejiang University of Technology, Hangzhou 310023, China; 3 China Aero Geophysical Survey and Remote Sensing Center for Land and Resources, Beijing 100083, China

**Keywords:** cold atom gravimeter, shipborne gravimeter, dynamic absolute gravimetry, coriolis effect modulation

## Abstract

Shipborne atomic gravimeter (SAG) is an instrument that can directly measure absolute gravity in dynamic environments. As a new type of gravity sensor, a standard method for evaluating its detailed performance has not been proposed and the detailed performance of SAG was rarely reported. In this paper, a system of dynamic gravity measurement, which was integrated with a home-made atomic gravimeter, is demonstrated, and a novel and simple method for testing the performance of SAG on the lake based on the modulated Coriolis effect is put forward. Firstly, in the state of ship mooring, a tilt modulation of the gravity sensor has been realized to make sure the Raman wave vector is parallel to the gravity axis. Moreover, a comparison between the measurement result of CG-5 and SAG has also been carried out to evaluate the accuracy of the SAG. Then, the Coriolis effect modulating experiment is carried out with various routes on lake to test its performance in dynamic environments. In the ship mooring state, the accuracy has been demonstrated to be 0.643 mGal. The internal consistency reliabilities are evaluated to be 0.8 mGal and 1.2 mGal under the conditions of straight line and circle navigation, respectively.

## 1. Introduction

Precise measurement of gravitational acceleration has applications in many fields, including precision measurement of fundamental physical constant [1], resources (oil, mineral deposits and natural gas) exploration [2], inertial navigation, etc. Some gravimeters are placed indoors for high-precision gravity observation for hydrological monitoring [3,4,5], earthquake forecasting [6], and volcanic activity monitoring [7]. There are also some gravimeters installed on carriers such as trucks, aircrafts, and ships for dynamic gravity measurement.

In terms of marine measurement, most marine gravity observations are based on relative gravimeters [8,9,10] and the internal consistency reliability of this kind of gravimeter is generally around 1 mGal. It has a drift, which is about 3 mGal per month. Performance evaluation of every marine relative gravimeter is often complicated [11,12]. Usually static tests would be carried out on land to evaluate the drift of the instrument. The dynamic performance evaluation requires a long-term testing at sea, which means a huge cost of resource and time. In general, the performance of the marine relative gravimeter is evaluated by the absolute gravimeter calibration, maritime gravity network measurement, repeated measurement lines, crossing point error analysis, multiple relative gravimeters comparison on the same ship, and long-term voyage measurement. The process generally lasts several years.

To make dynamic absolute gravity mapping more efficient, the cold atom absolute gravimeter has begun to play its role in recent years [13,14,15,16]. Interference characteristic of cold atomic matter waves are used to extract gravity information by accurately measuring the phase change of interference fringes caused by gravitational acceleration. Thanks to its working principle, the cold atomic gravimeter has almost no long-term drift [17]. As for the dynamic marine gravity measurement, the gravimeter system includes a gravity sensor, a control cabinet and an inertial stabilized platform [18]. Researchers of ONERA have implemented a shipborne system for dynamic measurement, which was based on the cold atomic gravimeter to measure the absolute gravity in 2018. The system has passed the maritime navigation survey line, and the internal consistency reliability of this measurement was less than 1 mGal [13]. In 2019, they have achieved an airborne dynamic absolute gravity measurement based on the same system with an internal consistency reliability of about 3 mGal [14].

As a new type of dynamic gravimeter, SAG has no standard method for performance evaluation yet. Here, we present a novel method for the performance testing of SAG on the lake. All gravity measurements in this paper were processed with the vibration compensation algorithm to obtain the gravity values [15,18]. Firstly, in the mooring environments, the tilt of gravity sensor was modulated to make sure that the Raman wave vector in the gravity sensor was parallel to the gravity axis. Then, a long-time measurement of about 12 h was carried out to evaluate the stability and the resolution. After that, the result of the above measurement would be compared with a relative gravimeter of CG-5. A difference of 0.2 mGal has been obtained between CG-5 and SAG measurements and an accuracy of 0.643 mGal of SAG has been obtained. Secondly, in sailing environments, it is hard to take a conventional survey line method for gravity measurement on a lake due to the limited lake area and the small changes of the gravity value of the lake area. Therefore, the velocity of the ship heading in an east-west direction was considered to modulate the Coriolis effect by changing the direction of the ship sailing, which would generate a large gravity fluctuation. Different sailing routes were designed on the lake for the Coriolis effect modulation experiment. In addition, three gravity check points were set up on the routes to validate the measured gravity values of the ship sailing states. An internal consistency reliability of 1.2 mGal of gravity measurements on the lake, which is based on shipborne atomic gravimeter in the state of ship sailing, can be realized after the Eötvös Effect correction. The home-made shipborne atomic gravimeter is expected to improve the efficiency of marine gravity mapping in the future.

## 2. Theory and Experimental Apparatus

The principle of cold atomic gravimeter is based on a Mach-Zehnder type atom interferometer. The ^87^Rb atoms are cooled and trapped in a vacuum after a 280 ms of three-dimensional magneto-optical trap loading and a 20 ms of polarization gradient cooling process, then about 5 × 10^7 87^Rb atoms at a temperature of 6 μK are obtained. A microwave pulse and a Raman π laser pulse are utilized to select the atomic state in the magnetic insensitive state of *F* = 2, *m_f_* = 0. With an interval of *T* = 20 ms and a duration of τ = 10 μs for π pulse, a sequence of π/2-π-π/2 Raman pulses are used to split, reflect and combine the atomic wave packets, and an atomic fluorescence detection with two PDs (photodiode) is used to detect the population of atoms in *F* = 2 and 1 states by the normalized detection method [19]. The gravitational *g_p_* measured by atomic gravimeter can be expressed as:

(1) $$g_{p}=\frac{\mathrm{acos}\left(\frac{2(P-A)}{C}\right)-\varphi_{vib}-\varphi_{other}+\alpha T^{2}}{k_{eff}T^{2}}$$

where *A* is the offset, *C* is the contrast, *P* is the transition probability, *α* is the chirp rate, *k_eff_* is the efficient wave vector of Raman laser, *φ_vib_* is the phase caused by vibration noise and *φ_other_* is the phase caused by other factors such as laser noise, magnetic noise and detection noise, respectively. As for the dynamic measurement of gravity, the Eötvös effect needs to be considered, which can be written as [13]:

(2) $$a_{corr}=2\Omega_{e}\cdot (e\times v_{ship})+v_{ship}{}^{2}\cdot R_{e}$$

where $\Omega_{e}$ is the angular velocity of the Earth’s rotation, e is the unit normal vector perpendicular to the survey location, $v_{ship}$ is the velocity of ship and $R_{e}$ is the radius of the Earth.

To show the effect of vibration correction intuitively, we use the calculated vibration correction phase to restore the results of atomic interference measurement. We calculate the chirp rate correction $\Delta_{\alpha }$ to correct *α* in the fringe, which is shown in Formula (3) [15,18]:

(3) $$\Delta_{\alpha }=\frac{\varphi_{vib}}{2\pi T^{2}}$$

**Figure 1:**
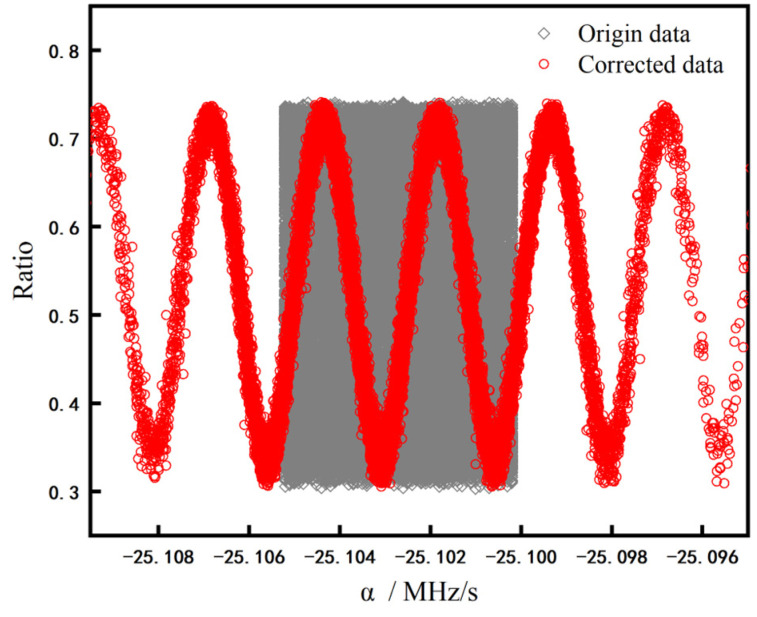
The signal of atomic interference fringe after vibration correction.

The corrected results are shown in Figure 1. The gray diamond points are the original population data, and the red round points are the corrected population data. There is no distinguishable fringe in the original data, while the fringe in the corrected data shows a good signal-to-noise ratio (SNR). Before the ship-borne test, the static performance of SAG was tested in the laboratory and in the field on a truck, respectively. The repetition rate of the gravimeter is 2 Hz. In the laboratory, the accuracy of the gravimeter is 14.2 μGal, and the sensitivity is about 0.2 mGal/Hz^1/2^. In the field on the truck, the accuracy of the gravimeter is 0.112 mGal and the internal consistency reliabilities of the gravimeter is 0.123 mGal. The sensitivity in static of the gravimeter is 1 mGal/Hz^1/2^ [20].

**Figure 2:**
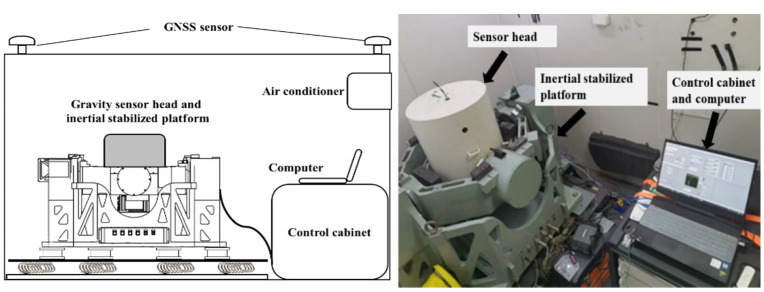
The experimental apparatus for shipborne gravity measurement system.

The experimental setup for shipborne gravity measurement is shown in Figure 2, which is installed in a standard container with the dimensions of 2.96 m × 2.44 m × 2.75 m. The shipborne atomic gravimeter is composed of a vacuum sensor, which provides an environment for atomic interference, and an inertial stabilized platform, which is used to maintain the vacuum sensor aligned with the gravity acceleration with a precision of 2.0 ″ and 5.4 ″ in roll and pitch angle, respectively. The control cabinet includes a laser system, a frequency chain, an electrical control and a data acquisition system [20,21].

A classical accelerometer (AS-803C3W1 from Japan) is installed directly in the inertial stabilized platform just under the vacuum sensor. It is a servo accelerometer built in two horizontal and vertical components in a case. The sensitivity of the accelerometer is 0.25 V/m/s^2^, the dynamic range is 140 dB (rms), the bandwidth is DC-250 Hz (−3 dB) and the full-scale range is ±40 m/s^2^. The Raman reflect mirror is need to install on the top of the accelerometer to acquire the ideal vibration acceleration measured by the accelerometer as much as possible. With a 0.05 Hz high pass filter and a time matching, the vibration data is used to correct the phase of the fringe. The two-stage shock absorber is applied to reject high-frequency vibration noise, and four air cushions are used to reject the low-frequency vibration noise, whose cut-off frequency is close to 5 Hz. Six wire rope shock absorbers are mainly used to resist the impact effect.

**Figure 3:**
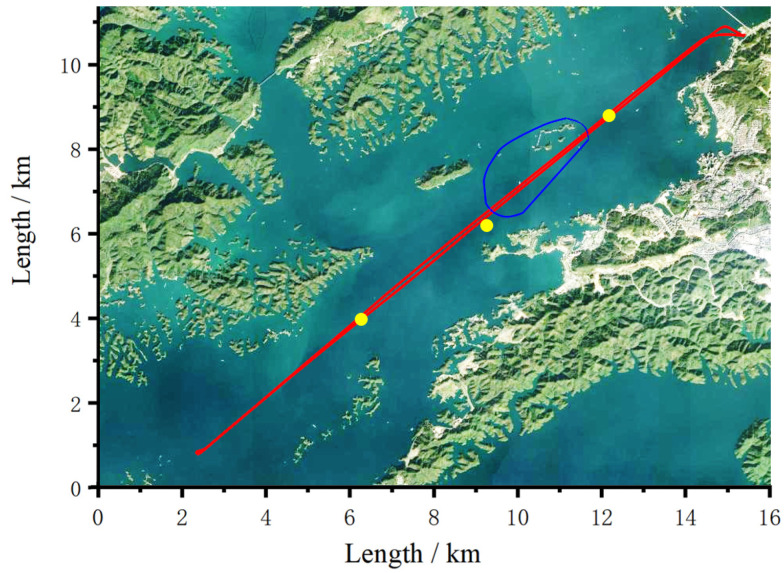
Routes of gravity survey on the Qiandao lake.

The gravity survey measurements were performed on the Qiandao lake with a ship sailing in two different routes, which are depicted in Figure 3. The red line and the blue circle depict the east-west straight route and the circle route, respectively. The velocity is about 12 km/h, which is almost the highest speed of the ship. The yellow points are checkpoints with an interval of about 4 km. The middle point deviated from the route by about 100 m due to a typhoon. After the sailing measurements, the ship anchored at every check point for a 20-min gravity measurement in ship mooring states. During the period of gravity measurement, the lake was not drained or stored, so that the gravity gradient correction caused by the change of height was not needed.

## 3. Results

**Figure 4:**
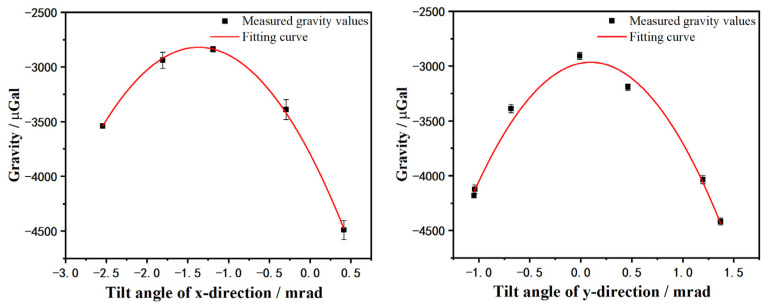
The results of tilt modulation experiments.

For the convenience of expression, we subtract a value $g_{0}$ from the gravity value in this paper to make the gravity value be a short number, and $g_{0}$ is 9.79246022 m/s^2^. The tilt modulation measurement with different tilt angles in x and y axes of the sensor has been realized on the lake. By fitting the measurement results on each axis with a parabolic model, the zero-tilt point of each axis was acquired. The angle fitting uncertainty is about 0.06 mrad, which causes about 2 μGal deviation of the absolute gravity value. In fact, such deviation can be ignored. The fitting results are shown in Figure 4.

**Figure 5:**
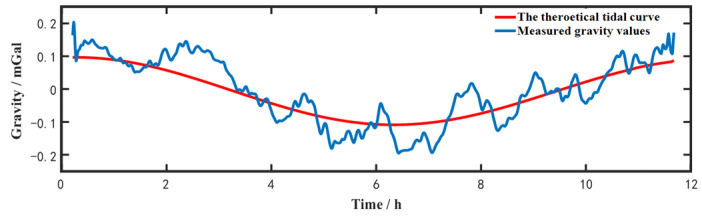
Gravity measurements in the state of ship mooring on the Qiandao lake.

In ship mooring environments, a 14-h gravity measurement with *T* = 20 ms was taken to evaluate the stability and the resolution of SAG. We adjust the measured gravity values to the same level with the theoretical tidal value, which are shown in Figure 5 below. The blue line is the gravity measurement data with a half hour low-pass filter, and the red curve is calculated by tide model. Obviously, the trend of measured gravity values obeys the theoretical tidal model.

**Figure 6:**
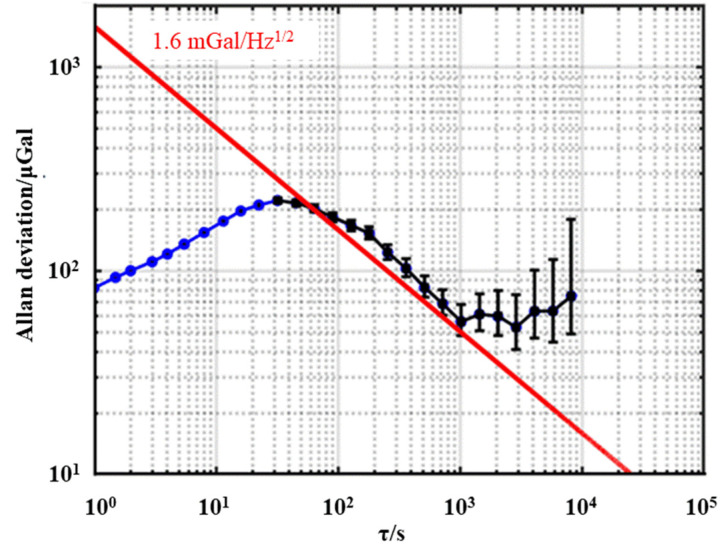
Allan deviation of measured gravity values in the state of ship mooring.

Figure 6 shows the Allan deviation of gravity measurement in mooring state, where the black points are fitted points and the red line is the fitted line. The sensitivity of shipborne atomic gravimeter is about 1.60 mGal/Hz^1/2^ and a resolution of 50 μGal could be reached with an integration time of 1000 s. In mooring environments, the systematic effects and the corrections were evaluated and listed in Table 1 [20].

To perform a further evaluation on the accuracy of gravity measurement result, a comparison with a high-precision absolute gravity reference value obtained by a relative gravimeter of CG-5 was conducted. The CG-5 gravimeter was set on the ground close to the ship, and the center of CG-5 is 2.77 m higher than the center of the gravity sensor. After the systematic effects correction in Table 1, a difference of 0.2 mGal has been obtained between CG-5 and SAG measurements.

**Figure 7:**
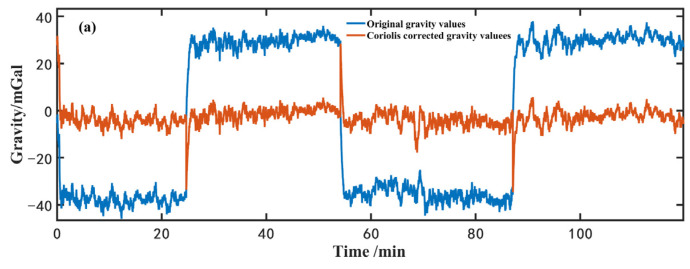

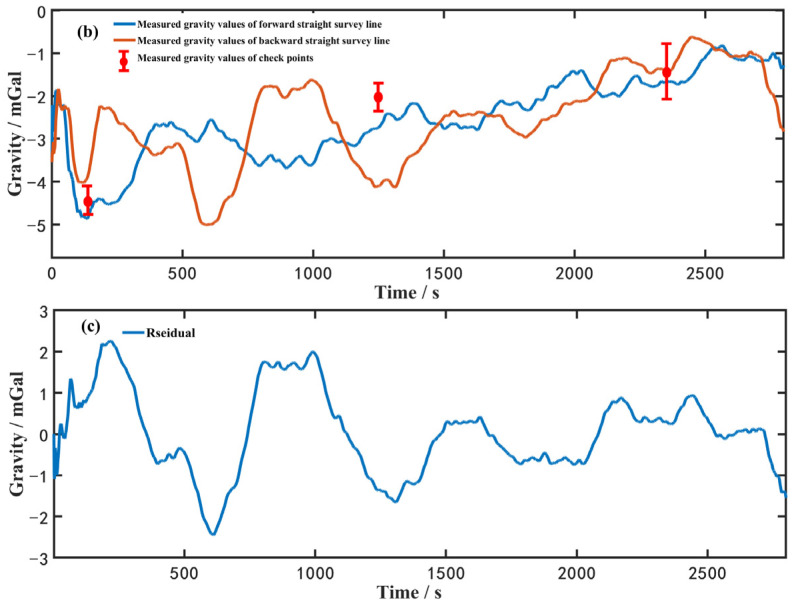
Two repeat gravity measurements in the ship sailing of straight survey line. Please see the detail explanation of the (**a**–**c**) in the above paragraph.

In sailing environments, the gravity measurements along the straight survey line with the velocity of 12 km/h were conducted four times, and the measurement results are shown in Figure 7. In Figure 7a, the blue curve shows the original gravity value *g_p_* before the Coriolis effect correction, and the orange curve shows the Coriolis corrected gravity value. There is a difference of 75 mGal between forward and backward routes due to the Coriolis change caused by the opposite ship heading. The accuracy of the ship velocity is below 0.03 m/s, and the accuracy of the ship’s sailing heading direction is below 3. The Coriolis noise is lower than 0.22 mGal. After correction, the measured gravity values almost remain at the same level. Figure 7b shows the results of the forward and backward measurements. The red points in Figure 7b are the measured gravity values at checkpoints, which are consistent with the results of the ship sailing measurement. Figure 7c is the residuals of two repeat measurements with a low-pass Butterworth filter of time constant, 150 s. The peak-to-peak value of residuals is around ±2.0 mGal and the standard deviation is about 0.8 mGal.

**Figure 8:**
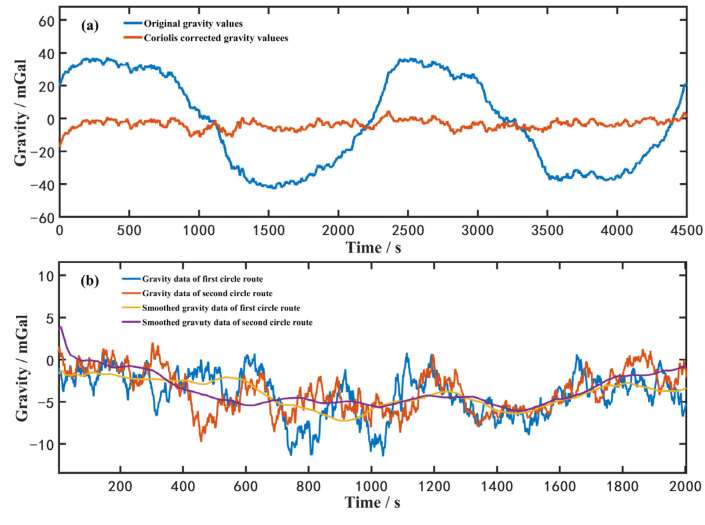

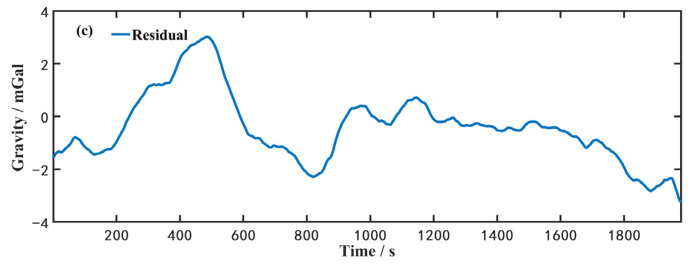
Two gravity measurements in ship sailing state for circle routes. Please see the detail explanation of the (**a**–**c**) in the above paragraph.

Then, we perform gravity measurements in circle routes twice, and the results are shown in Figure 8. In the circle route measurements, the Coriolis correction varies with the direction of ship heading. All the results of gravity measurements were processed with a low-pass Butterworth filter of time constant 150 s. The blue curve in Figure 8a is the original measured gravity values and the orange curve is the Coriolis-corrected values of the circle route measurements. The blue and orange curves in Figure 8b are Coriolis-corrected results, and the yellow and the purple curves are the filtered data. The curve in Figure 8c is the residuals of two measurements. A very good reproducibility is obtained between two measurements with a standard deviation on the difference equal to 1.2 mGal and a peak-to-peak value of measured gravity about ±2 mGal. Additionally, we compared the gravity value at two cross points between the straight line and circle route, and find out that the residuals were 0.1 mGal and 1.9 mGal, respectively.

## 4. Conclusions

In this paper, a system of shipborne gravity measurement has been integrated based on a home-made atomic gravimeter; we proposed a novel and simple method to evaluate the performance of this instrument on the lake. To be compared with a relative gravimeter of CG-5 in a ship mooring state, an external coincidence accuracy of 0.2 mGal was obtained. To further evaluate the internal consistency reliability, we performed gravity measurements in the ship sailing state with the velocity of 12 km/h in a straight-line and modulate the ship heading direction to change the Coriolis correction. After the Coriolis correction, the internal consistency reliability is 0.8 mGal in straight line measurements and 1.2 mGal in circle route measurements. By this method, we can evaluate instruments’ performance with small ships on small water area, which reduce the cost greatly, compared with the traditional marine gravity measurement based on relative gravimeters. The overall evaluation time is generally about one week, which will greatly improve the evaluation efficiency.

## Figures and Tables

**Table 1:** Systematic effects and corrections table for SAG in mooring environments.

	Bias/μGal	Uncertainty/μGal
Coriolis effect	0.0	39.0
Two-photon laser shift effect	−545.4	49.6
Laser frequency	−5.5	1.1
Atomic clock frequency	0.0	1.0
RF phase shift	−131.5	10.8
Self gravitation effect	−2.7	0.1
Laser sideband effect	220.0	639.7
Other correction	0.2	2.0
Total	−464.9	642.9

## Data Availability

Data underlying the results presented in this paper are not publicly available at this time but may be obtained from the authors upon reasonable request.
